# Hyaluronic acid-coated polymeric micelles with hydrogen peroxide scavenging to encapsulate statins for alleviating atherosclerosis

**DOI:** 10.1186/s12951-020-00744-w

**Published:** 2020-12-07

**Authors:** Dan Mu, Jianhui Li, Yu Qi, Xuan Sun, Yihai Liu, Song Shen, Yuyu Li, Biao Xu, Bing Zhang

**Affiliations:** 1 Department of Radiology, Affiliated Nanjing Drum Tower Hospital of Nanjing University Medical School, 321 Zhongshan road, Nanjing, 210008 Jiangsu China; 2 Department of Cardiology, Nanjing Drum Tower Hospital, Clinical College of Nanjing Medical University, Nanjing, 210008 China; 3 Department of Cardiology, Affiliated Nanjing Drum Tower Hospital of Nanjing University Medical School, Nanjing, 210008 China; 4 Department of Cardiology, Nanjing Drum Tower Hospital, Medical School of Nanjing University, Nanjing, 210008 China; 5 State Key Laboratory of Pharmaceutical Biotechnology, Nanjing University, 321 Zhongshan road, NanjingJiangsu, 210023 China; 6 Institute of Brain Science, Nanjing University, 321 Zhongshan road, Nanjing, 210008 Jiangsu China

**Keywords:** Simvastatin, ROS-responsive, Hyaluronic acid, Macrophages, Antioxidative stress

## Abstract

Inflammation and oxidative stress are two major factors that are involved in the pathogenesis of atherosclerosis. A smart drug delivery system that responds to the oxidative microenvironment of atherosclerotic plaques was constructed in the present study. Simvastatin (SIM)-loaded biodegradable polymeric micelles were constructed from hyaluronic acid (HA)-coated poly(ethylene glycol)-poly(tyrosine-ethyl oxalyl) (PEG-Ptyr-EO) for the purpose of simultaneously inhibiting macrophages and decreasing the level of reactive oxygen species (ROS) to treat atherosclerosis. HA coating endows the micelle system the ability of targeting CD44-positive inflammatory macrophages. Owing to the ROS-responsive nature of PEG-Ptyr-EO, the micelles can not only be degraded by enzymes, but also consumes ROS by itself at the pathologic sites, upon which the accumulation of pro-inflammatory macrophages is effectively suppressed and oxidative stress is alleviated. Consequently, the cellular uptake experiment demonstrated that SIM-loaded HA-coated micelles can be effectively internalized by LPS-induced RAW264.7 cells and showed high cytotoxicity against the cells, but low cytotoxicity against LO2 cells. In mouse models of atherosclerosis, intravenously SIM-loaded HA-coated micelles can effectively reduce plaque content of cholesterol, resulting in remarkable therapeutic effects. In conclusion, the SIM-loaded micelle system provides a promising and innovative option against atherosclerosis.
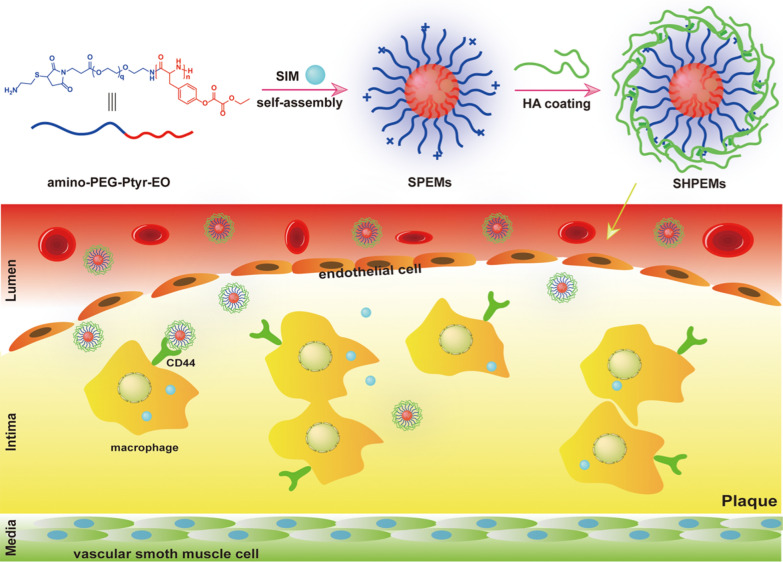

## Introduction

Atherosclerosis is a chronic, systemic inflammatory disease of the large and medium-sized arteries, characterized by the development of plaques due to the retention of lipids in the artery wall and infiltration of leukocytes, which can lead to life-threating events such as myocardial infarction and stroke [1–3]. The inflammatory area recruits monocytes, differentiating into macrophages. Upon ingestion of low-density lipoprotein (LDL), macrophages would die or even lead to cellular rupture, recruiting additional immune cells to inflammatory area [4]. Due to the decisive role and abundance in the progression of plaque, macrophages could achieve targeting capacity of plaques. CD44 is one of the specific biomarkers of macrophages and widely expressed on multiple cell types. In inflammatory area, CD44 is upregulated and functionally activated on vascular endothelial, smooth muscle and inflammatory cells. Furthermore, CD44 modulates leukocyte adhesion, migration, and functional phenotype [5]. All these facts make CD44 perfect for a potential therapeutic target of local inflammation at the site of lesion.

Statins are widely orally prescribed serum LDL cholesterol-lowering drugs, raising the expression of LDL receptor in hepatocytes [6]. Clinical data has shown that orally administered statins can reduce the risk of cardiovascular disease (CVD) and attenuate the development of atherosclerotic plaques [7]. Statins exhibit pleiotropic properties via reductase derived metabolites blockade, including antioxidant effects, anti-inflammatory effects and multiple vasculoprotective effects on endothelial cells, vascular smooth muscle cells and monocytes [8–10]. However, considering the low systemic bioavailability, increasing the oral statin dose to attain higher plasma concentrations is not feasible due to the dose-dependent onset of adverse effects such as hepatoxicity and myopathy [3, 6]. In addition, it was reported that high-dose statins probably induced body weight loss and abnormal serum biomarkers [11]. All these imperfections hinder the further application of statins in the clinical practice.

Increasing researches have demonstrated that the specific delivery of statins to inflammatory atherosclerotic plaques might optimize the therapeutic effects [12–16]. Nanocarriers can target plaques by direct infiltration via the injured endothelium or the dysfunctional neovessels in the adventitia [17]. In addition, nanocarriers administered via intravenous or intraperitoneal injection can be endocytosed by circulating phagocytes, followed by translocation to atherosclerotic lesions by cellular recruitment and infiltration. Whereas nanocarriers only serve as vehicles for targeted delivery of different therapeutics to atherosclerotic plaques in most studies, recent progress also indicated that nanocarriers with intrinsic antioxidant and anti-inflammatory activities are promising next-generation therapies for the treatment of atherosclerosis and other inflammatory diseases [18]. Andrographolide-loaded micelles that responds to the oxidative microenvironment of atherosclerotic plaques was constructed based on the block copolymer of poly(ethylene glycol) and poly(propylene sulphide) (PEG-PPS) for the purpose of simultaneously decreasing inflammatory response and the level of reactive oxygen species (ROS) to treat atherosclerosis [19]. Andrographolide exhibits great anti-inflammatory activity and the antioxidant activity of PEG-PPS was endowed by the thioether in the hydrophobic block PPS. Another research has reported a novel dual ROS-sensitive and CD44 receptors targeting nanocarriers composed of oligomeric hyaluronic acid-2′-[propane-2, 2-diyllbls (thio)] diacetic acl-hydroxymethylferrocene (HASF) [20]. Curcumin was encapsulated into HASF and provided potent anti-inflammatory and antioxidant effects. In addition, 2′-[propane-2, 2-diyllbls (thio)] diacetic acl (TKL) and hydroxymethylferrocene (Fc) acted as ROS-responsive linkers and also enhanced antioxidant effects. Another ROS-responsive delivery system combining anti-inflammatory and antioxidant effects has been constructed based on arylboronic esters-modified amphiphilic oxidation-sensitive chitosan oligosaccharide (Oxi-COS) [4]. Except for the above, peroxalate ester is another ROS-responsive bonds and has been used to establish drug delivery systems [21–23]. However, as far as the information goes, it has not been reported that peroxalate esters are used to achieve the combination of anti-oxidation and anti-inflammation in the treatment of atherosclerosis.

**Scheme 1.**
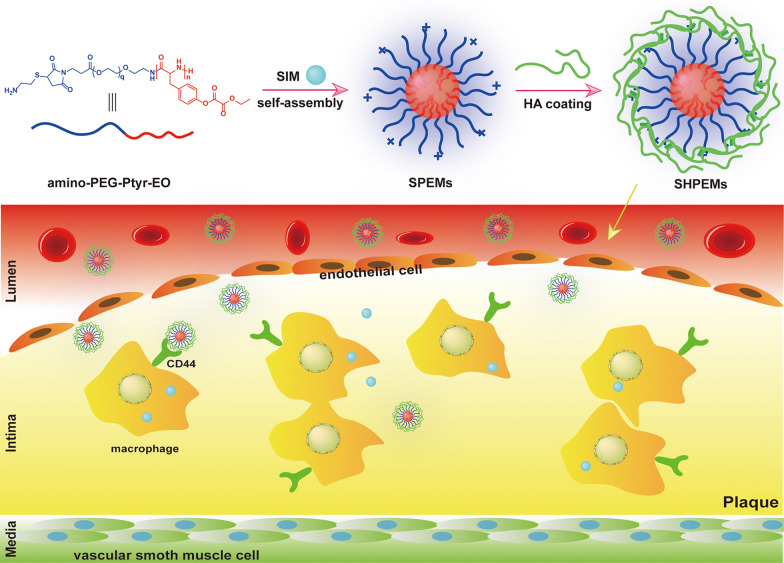
The preparation route and targeted delivery process of SIM from the SHPEMs. SIM: simvastatin; HA: hyaluronic acid; SPEMs: SIM-loaded amino-PEG-Ptyr-EO micelles; SHPEMs: SIM-loaded HA-coated amino-PEG-Ptyr-EO micelles

Herein, SIM-loaded hyaluronic acid (HA)-coated biodegradable polymeric micelles assembled from an amphiphilic diblock copolymer poly(ethylene glycol)-poly(tyrosine-ethyl oxalyl) (PEG-Ptyr-EO) were established for atherosclerosis treatment. The preparation route of SIM-loaded polymeric micelles was depicted in Scheme 1. SIM-loaded polymeric micelles were prepared by self-assembly and HA coating on the surface of micelles was achieved by electrostatic interaction. The hydrophobic block Ptyr-EO can react with H_2_O_2_ and this process takes place only at the pathologic sites where the H_2_O_2_ concentration is far above that in normal tissues, and thereby the drug delivery efficiency and therapeutic efficacy may be dramatically increased. Furthermore, SIM and the polymers serve to reduce inflammation and H_2_O_2_ levels, respectively, both of which are essential in the formation and progression of atherosclerosis. It is anticipated that, SIM and the drug carrier itself may exert a synergistic effect for effective treatment of atherosclerosis.

## Methods

### Materials

Maleimide poly(ethylene glycol) amine (Mal-PEG-NH_2_, 5000 Da) was obtained from Xi’an Ruixi Biological Technology Co., Ltd. Hyaluronic acid (HA, 40–100 kDa), cysteamine hydrochloride and ethyl oxalyl chloride were purchased from Shanghai McLean Biochemical Technology Co., Ltd. L-Tyrosine *N*-carboxyanhydride (Tyr-NCA) was brought from Shanghai Bide Pharmaceutical Technology Co., Ltd. Lipopolysaccharide (LPS) was brought from Sigma-Aldrich. Cy5 NHS ester was purchased from Shanghai Yuanye Biotechnology Co., Ltd. 2′, 7′-Dichlorofluorescin diacetate (DCFH-DA) was obtained from Beyotime Institute of Biotechnology. Anhydrous *N*, *N*-dimethylformamide (DMF) and dichloromethane (DCM) were purchased from Energy Chemical. Other solvents, including dimethylsulfoxide (DMSO), tetrahydrofuran (THF) and triethylamine (TEA) were brought from Greagent and used as received.

### Synthesis of Mal-PEG-Ptyr

Mal-PEG-Ptyr was synthesized according to the report [24, 25]. Briefly, Mal-PEG-NH_2_ (1.0 g, 0.2 mmol) and Tyr-NCA (1.0 g, 4.8 mmol) were dissolved in anhydrous 15 mL DMF. The reaction was performed at 35 °C under N_2_ for 72 h. The reaction mixture was concentrated under vacuum and poured into excess cold diethyl ether for five times. The precipitation was collected and dried under vacuum.

^1^H NMR (500 MHz, DMSO-*d*_*6*_) δ: 9.09 (s, 23.81 H, −O***H***), 7.92 (m, 21.93 H, −CON***H***−), 7.08−6.56 (m, 105.02 H, −C_6_***H***_4_−), 4.43 (m, 20.44 H, −C***H***−), 3.56 (m, 450.00 H, −C***H***_2_C***H***_2_−), 2.89–2.59 (m, 44.80 H, −C***H***_2_C_6_H_4_−).

### Synthesis of Mal-PEG-Ptyr-EO

Mal-PEG-Ptyr (0.5 g) was dissolved in anhydrous DMF. TEA (0.6 mL) was added into DMF solution. Ethyl oxalyl chloride (0.3 g, 2.2 mmol) in DCM was added dropwise in ice bath. The reaction was stirred for 24 h. After that, the reaction mixture was filtrated and washed by saturated NaCl solution for three times. The organic phase was collected and poured into excess cold diethyl ether for three times. The precipitation was collected and dried under vacuum.

^1^H NMR (500 MHz, DMSO-*d*_*6*_) δ: 7.49−6.73 (m, 85.98 H, −C_6_***H***_4_−), 4.31 (m, 40.11 H, −C***H***_2_CH_3_), 3.56 (m, 450.00 H, −C***H***_2_C***H***_2_−), 1.30 (m, 63.53 H, −CH_2_C***H***_3_).

### Synthesis of amino-PEG-Ptyr-EO

Mal-PEG-Ptyr-EO (0.3 g) was dissolved in 10 mL DMF with cysteamine hydrochloride (0.1 g) added. The reaction was stirred at room temperature for 24 h. After that, the mixture was diluted with DCM, washed with saturated NaCl solution for three times and dried with anhydrous Na_2_SO_4_. After concentration, the mixture was poured into cold diethyl ether. Amino-PEG-Ptyr-EO was obtained by filtration and drying under vacuum. To label amino-PEG-Ptyr-EO with fluorescence dye, 10 mg of Cy5 NHS ester was reacted with 50 mg of amino-PEG-Ptyr-EO to generate Cy5-PEG-Ptyr-EO.

^1^H NMR (500 MHz, DMSO-*d*_*6*_) δ: 7.44 − 6.81 (m, 91.77 H, −C_6_***H***_4_−), 4.32 (m, 40.21 H, −C***H***_2_CH_3_), 3.56 (m, 450.00 H, −C***H***_2_C***H***_2_−), 1.29 (m, 61.77 H, −CH_2_C***H***_3_).

### H_2_O_2_ degradation

Mal-PEG-Ptyr-EO was dissolved completely in DMSO-*d*_6_ with addition of 50 mM H_2_O_2_ in a nuclear magnetic tube. The tube was incubated at 37 °C for 30 min, 60 min and 120 min. The chemical structure was characterized by ^1^H NMR. The amount of H_2_O_2_ was detected after incubation for 30 min, 60 min and 120 min by hydrogen peroxide assay kit (Beyotime).

### Preparation of micelles

Before preparing micelles, the molecular weights and their corresponding distribution of polymers were measured by the gel permeation chromatography system (GPC, Waters). THF was used as the eluent at a low flow rate of 1.0 mL/min. The standard curve was constructed by monodispersed polystyrene standard. The critical micelle concentrations (CMC) for the block copolymers were determined using pyrene as the probe [26]. Briefly, a constant concentration of pyrene (5.0 × 10^–7^ mol/L) was incubated with varying concentrations of lyophilized block copolymer (0.05–51.2 μg/mL) in phosphate buffer solution (PBS) at 37 °C for 36 h. All samples were run with a total sample volume of 1.2 mL in a quartz cuvette. The spectrophotometer (Hengping F9600) was used to record the excitation spectra for each polymer concentration at 338 and 333 nm. A plot of the *I*_338_/*I*_333_ ratio versus log of the block copolymer concentration was drawn to calculate the CMC.

Micelles based on amino-PEG-Ptyr-EO were prepared by dialysis method. Briefly, 10 mg amino-PEG-Ptyr-EO dissolved in 1 mL DMSO completely was placed into a dialysis tube (*M*_w_ 3500) and dialyzed against pure water for 24 h. Amino-PEG-Ptyr-EO micelles (PEMs) were obtained by lyophilization. HA-coated PEMs (HPEMs) were prepared by electrostatic attraction. Briefly, PEMs suspension was slowly added to a HA solution under vigorous stirring at room temperature. The mass ration of HA to PEMs and the concentration of HA were further investigated to obtain the optimum formulation. The method to prepare Cy5-labeled micelles was as mentioned above, except that amino-PEG-Ptyr-EO was replaced with amino-PEG-Ptyr-EO/Cy5-PEG-Ptyr-EO (95/5, w/w).

### Characterization of micelles

The size distribution and Zeta potential of micelles were recorded by dynamic light scattering (DLS, Malvern Zetasizer). The morphologies of micelles were investigated by transmission electron microscopy (TEM, JEM-2100). In brief, a drop of micelle suspension was placed onto the surface of the copper grid. After stained for 5 min, the suspension on the copper grid was absorbed by filter paper. The TEM photos were taken after air-drying.

### Drug encapsulation and release

SIM-loaded PEMs (SPEMs) were prepared by dialysis method. Briefly, 100 mg amino-PEG-Ptyr-EO and 10 mg SIM were dissolved in 2 mL DMSO completely. Then, the mixture was placed into a dialysis tube (*M*_w_ 3500) and dialyzed against pure water for 48 h to remove DMSO and unloaded SIM. After that, the suspension was filtrated through 0.45 μm syringe filter. Afterwards, SIM-loaded HPEMs (SHPEMs) were obtained by HA coating with 0.5 mg/mL HA solution with the mass ratio of 3/1 (HA to SPEMs). Besides, SIM-loaded Mal-PEG-Ptyr micelles (SPMs) were prepared by the same method and used in drug release experiments. SHPEMs were incubated in PBS (pH 7.4) and DMEM with 10% FBS for 16 days. The average size was measured at predetermined time points.

To determine the drug loading efficiency (DLE) and drug loading content (DLC), a known amount of SPEMs was dissolved in 1 mL of THF. This solution was then analyzed by an ultraviolet (UV) spectrophotometer at 238 nm with PEMs suspension as the control [27]. The drug concentration in the solution was calculated based on the standard curve. DLE and DLC were obtained in accordance with the following formulae:

1 $$DLC \%=\frac{weight\,of\,SIM\,loaded}{weight\,of\,SIM\,loaded\,micelles}\times 100 \%$$

2 $$DLE \%=\frac{weight\,of\,SIM\,loaded}{weight\,of\,SIM\,in\,feeding}\times 100 \%$$

The drug release was conducted based on SPEMs and SPMs. Briefly, 1 mL of SPEMs or SPMs suspension with the same SIM amount were placed into the dialysis bag (*M*_w_ 3500) suspended in 20 mL PBS (pH 7.4) containing 0.2% SDS with or without 10 mM H_2_O_2_. The sealed vials were shaken at 37 °C for 72 h. At predetermined time intervals, the release medium was withdrawn to measure the amount of SIM by UV method and 20 mL fresh medium was added. The experiment was performed in triplicates. After the experiment, the residues of SHPEMs were collected to measure the size distribution and morphology by DLS and TEM.

### Cell culture

RAW264.7 and LO2 cells were employed to evaluate the cellular uptake and cytotoxicity against SIM and SIM-loaded micelles. RAW264.7 cells, highly expressed CD44 receptors, were cultured in Dulbecco’s modified eagle’s medium (DMEM) containing 10% FBS, 100 mg/mL of penicillin and 100 mg/mL of streptomycin at 37 °C in an incubator with 5% CO_2_ humid atmosphere. To induce inflammatory reaction, RAW264.7 cells were simulated with 100 ng/mL LPS for 24 h. LO2, negatively expressed CD44 receptors, cells were cultured in RPMI 1640 supplemented with 10% FBS, 100 mg/mL of penicillin and 100 mg/mL of streptomycin at 37 °C in an incubator with 5% CO_2_ humid atmosphere.

### Cellular uptake

LPS-simulated RAW264.7 cells were reported to highly express CD44 receptors that can specifically bind HA ligands. However, LO2 cells were reported to negatively express CD44 receptors [28]. Therefore, RAW264.7 and LO2 cells were seeded into glass plates with the density of 1.0 × 10^5^ cells/plate. After incubation for 12 h, the culture medium was replaced with fresh medium containing Cy5-labeled PEMs or HPEMs with or without HA pretreatment. After incubation for another 2 h, cells were washed with PBS for two times and fixed with 4% paraformaldehyde for 15 min. After stained with DAPI for 10 min, fluorescence images of cells were obtained using a confocal laser scanning microscope (CLSM, Leica).

### Cytotoxicity

The cytotoxicity of SIM and SIM-loaded micelles against RAW264.7 or LO2 cells was measured by MTT assay. Briefly, LPS-simulated RAW264.7 cells were seeded at a density of 4 × 10^3^ cells/well in 96-well plates. When the cells reached 80% confluence, blank micelles, free SIM, SPEMs or SHPEMs at different SIM concentrations (0.25, 0.5, 1.0, 2.0, 4.0, and 8.0 μg/mL) or different polymer concentrations (50, 100, 200, 300, 400 and 500 μg/mL) in DMEM were added and cultured for 24 h. LO2 cells were seeded and cultured as the same method, and treated with free SIM and SHPEMs (SIM: 0.25, 0.5, 1.0, 2.0, 4.0, and 8.0 μg/mL) for 24 h. To evaluate the time-dependent cytotoxicity, cells were incubated with free SIM and SHPEMs (SIM: 4.0 μg/mL) for 24 h, 48 h and 72 h. To survey the influence of HA coating on the cytotoxicity, cells were incubated with free SIM, SPEMs and SHPEMs at the same SIM concentration (8.0 μg/mL) for 4 h. Then, the culture medium was replaced with fresh medium and cells were incubated for another 20 h. 10 μL of 5 mg/mL MTT solution was added to each well and the cells were incubated for another 4 h. Subsequently, the medium was carefully removed and 100 μL of DMSO was added to each well. The absorbance of each well was recorded on a microplate reader at the wavelength of 562 nm. The experiment was performed in triplicates.

3 $$Cell\,viability \%=\frac{{OD}_{sample}-{OD}_{blank}}{{OD}_{control}-{OD}_{blank}}\times 100 \%$$

where OD_sample_ means the OD value from wells treated with drugs, OD_control_ from wells treated with DMEM medium and OD_blank_ from wells without cells but culture medium.

### ROS generation

DCFH-DA was used to evaluate the generation of intracellular ROS by fluorescence imaging and flow cytometry. In brief, RAW264.7 and LPS-induced RAW264.7 cells were seeded in a 6-well plate and cultured for 12 h at 37 °C. Next, cells were incubated with DCFH-DA (10 µM) for 30 min. Then the cells were washed with PBS and incubated with different samples respectively (PBS, free SIM, HPEMs and SHPEMs). After incubation for another 12 h, the cells were washed with PBS to remove free samples, following by observation using a fluorescence microscopy and analysis by flow cytometry.

### Aminals treatment

Apolipoprotein E-deficient (ApoE^−/−^) mice (six-week old) were obtained from the Nanjing University in Nanjing, China. All the animal care and experimental protocols were carried out with review and approval from the Laboratory Animal Welfare and Ethics Committee of the Nanjing University. ApoE^−/−^ mice were fed with a high-fat diet for 8 weeks and treated once a week with saline, free SIM, SPEMs and SHPEMs at a dose of 30 mg/kg for SIM.

### ORO and H&E staining

After treatment for 4 weeks, the mice were sacrificed, and the aortas and major organs were harvested. Aortas, from the heart to the iliac bifurcation, were fixed by perfusion with paraformaldyhyde (4% in PBS). After removing the periadventitial tissue, aortas were dissected longitudinally and stained with Oil Red O (ORO) to quantify the lesion areas of the plaques by NIH ImageJ software. Besides, sections of the major organs including heart, liver, spleen, lung, and kidney were also analyzed by hematoxylin–eosin (H&E) staining.

## Results and discussion

### Synthesis of polymers

**Scheme 2:**
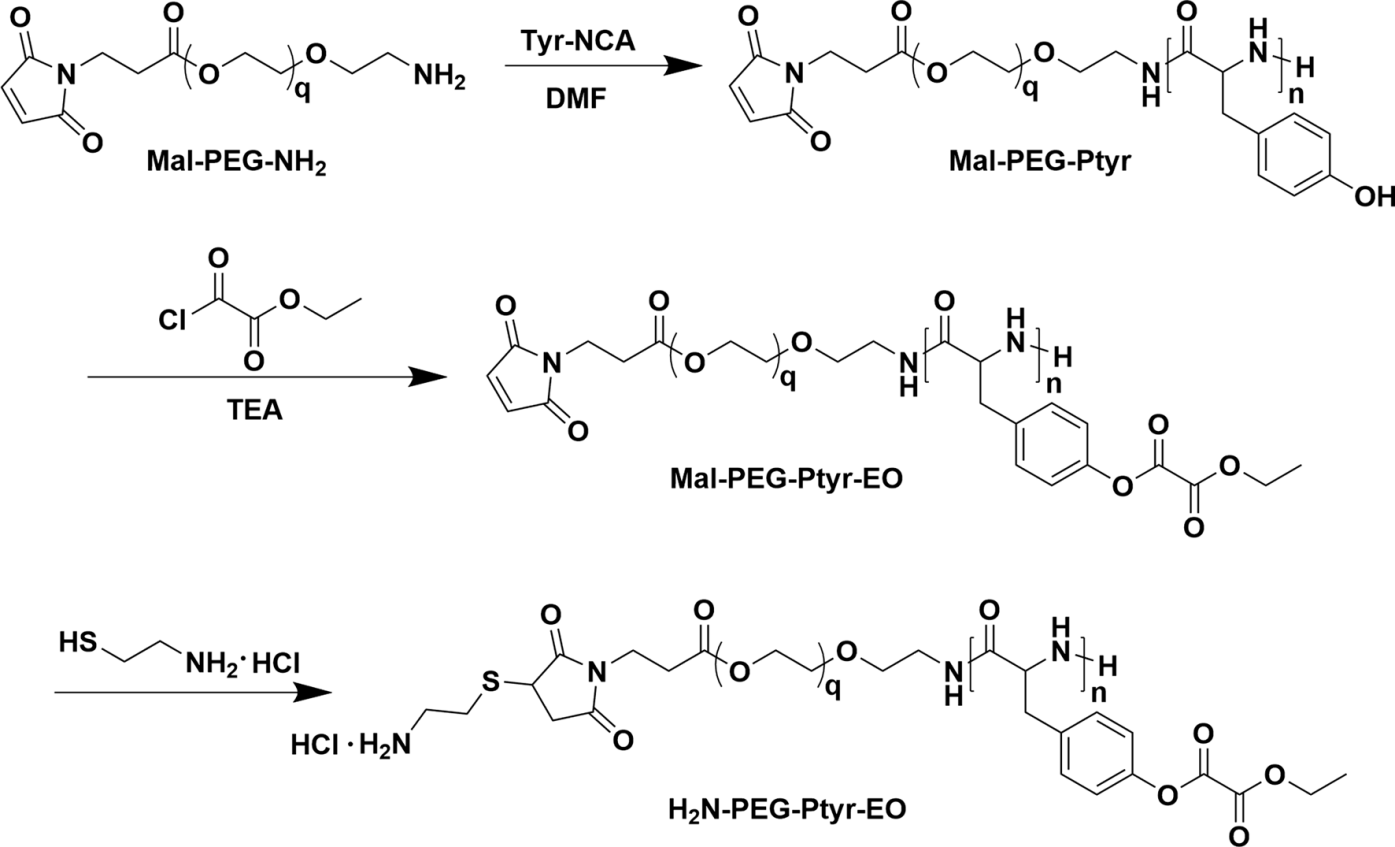
Schematic depiction of the synthetic route of amino-PEG-Ptyr-EO

As shown in Scheme 2, Mal-PEG-Ptyr copolymers were obtained via the ring-opening polymerization of tyrosine *N*-carboxyanhydride (Tyr-NCA) using Mal-PEG-NH_2_ as the initiator (Scheme 1). The ^1^H NMR spectrum of Mal-PEG-Ptyr displays the characteristic signals of PEG (peak ***a***, δ 3.56) and phenolic hydroxyl groups (peak ***f***, δ 9.09) (Additional file 1: Figure S1A). Other signals of Ptyr were labeled and attributed to benzene (peak ***d*** and ***e***, δ 6.96 and 6.60), methylene (peak ***c***, δ 2.84–2.66) and methyne (peak ***b***, δ 4.43), respectively. The integral area of these signal peaks was calculated and listed in Additional file 1: Figure S1A. The *M*_n_ of Mal-PEG-Ptyr block copolymers was calculated by comparing the integrals of peak ***a*** and peak ***c***, or peak ***e***. The *M*_n_ of Mal-PEG-Ptyr was also determined by GPC as shown in Table 1 and the trace of GPC was demonstrated in Additional file 1: Figure S2. GPC measurements suggested that Mal-PEG-Ptyr had a narrow molecular weight distribution.

**Table 1:** Molecular weight of copolymers

Copolymer	*M*_n_ (kg/mol)		*M*_w_/*M*_n_^b^
	^1^H NMR^a^	GPC^b^	
Mal-PEG-Ptyr	8.3–9.2	10.6	1.21
Mal-PEG-Ptyr-EO	10.7–11.3	13.2	1.25

^a^Calculated from ^1^H NMR (DMSO-*d*_6_); ^b^determined by GPC

Mal-PEG-Ptyr-EO was synthesized by conjugation of tyrosine units with ethyl oxalyl chloride. The ^1^H NMR spectrum of Mal-PEG-Ptyr-EO was exhibited in Additional file 1: Figure S1B. The emerging signals assigned to ethyl oxalyl esters were listed and labeled as methylene (peak ***g***, δ 4.31) and methyl groups (peak ***h***, δ 1.30) that indicated the successful synthesis of Mal-PEG-Ptyr-EO. The integrals of peak ***g*** and ***h*** were calculated and compared with these of peak ***a***, suggesting that ca. 20 tyrosine units were modified by ethyl oxalyl chloride, which was evidenced by the increase of the molecular weight of Mal-PEG-Ptyr-EO in Table 1. Notably, after modification, the positions of signals assigned to Ptyr had changed (labeled as peak ***b’***, ***c’***, ***d’*** and ***e’***). At last, cysteamine was conjugated with Mal-PEG-Ptyr-EO to give amine-PEG-Ptyr-EO. The ^1^H NMR spectrum was demonstrated in Additional file 1: Figure S1C. The emerging peak ***i*** (δ 3.17) indicated the successful conjugation.

### H_2_O_2_ degradation

It has been reported that peroxalate ester can be oxidized rapidly by H_2_O_2_ to generate CO_2_ [23]. Hence, the polymer with peroxalate ester linkages serve as a new family of biocompatible and biodegradable materials, which is applied to deliver therapeutic drugs to inflammation sites or tumor tissues that overproduce ROS species. Theoretically, Mal-PEG-Ptyr-EO can be oxidized into Mal-PEG-Ptyr, CO_2_ and alcohol as shown in Additional file 1: Figure S3A. To prove it, Mal-PEG-Ptyr-EO was incubated with 50 mM H_2_O_2_ for different time and the chemical structures of polymers were monitored as shown in Additional file 1: Figure S3B. After incubation for 30 min, the signal peak ***h*** (δ 1.30) turned into peak ***h’*** (δ 1.21) and the signal of peak ***g*** decreased, indicating the generation of alcohol. After incubation for 60 min and 120 min, peak ***g*** disappeared completely. Besides, peak ***g’*** was probably hidden by the signal of PEG, which makes it cannot be observed. The degradation of Mal-PEG-Ptyr-EO by H_2_O_2_ was also proved by the consumption of H_2_O_2_ as shown in Additional file 1: Figure S4. In the first 60 min, H_2_O_2_ was gradually consumed. However, in the second 60 min, the amount of H_2_O_2_ was almost the same, indicating complete oxidation of Mal-PEG-Ptyr-EO.

### Micelle preparation and characterization

**Fig. 1:**
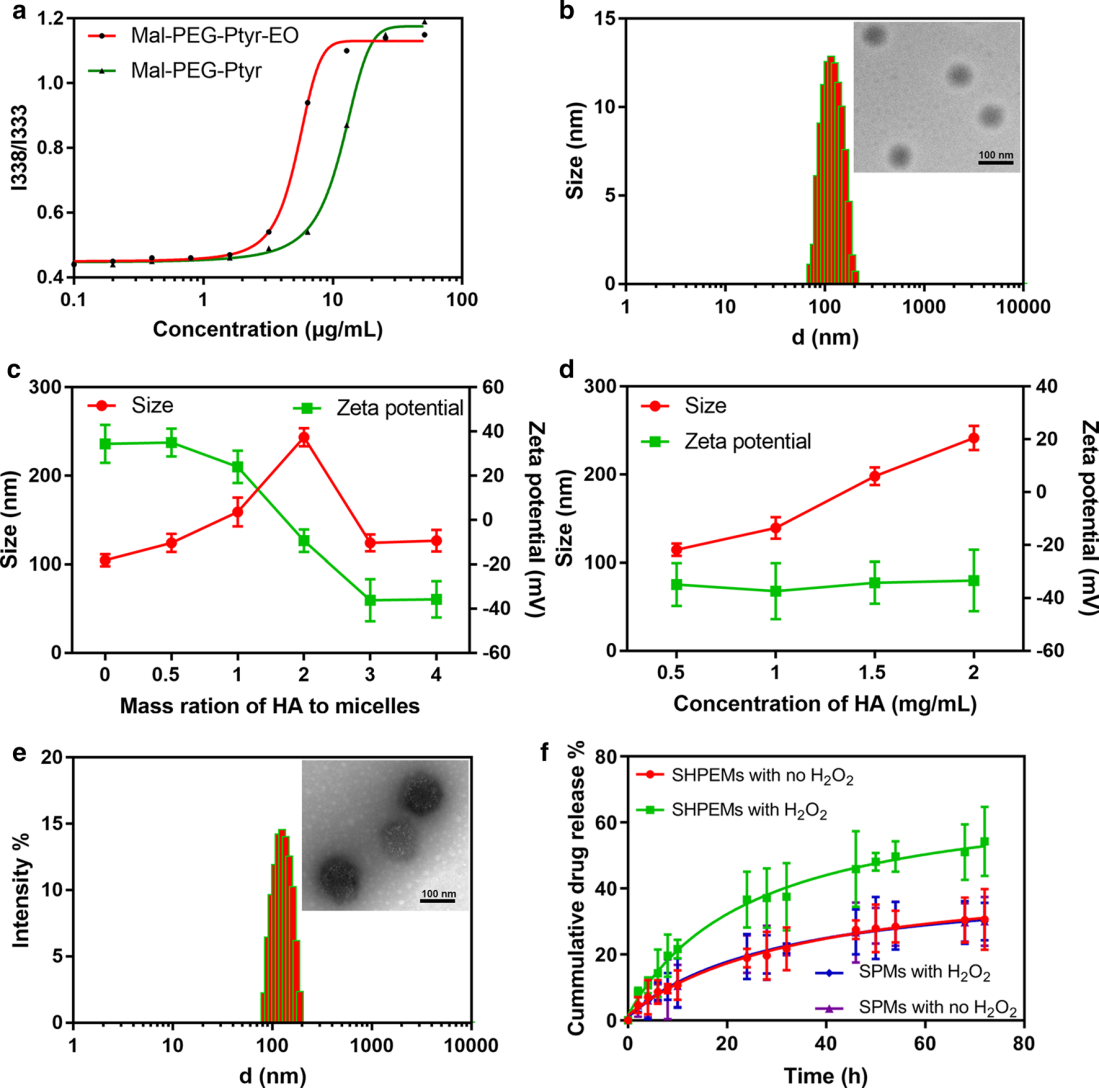
Determination of CMC of Mal-PEG-Ptyr and Mal-PEG-Ptyr-EO (**a**); size distribution and morphology of amino-PEG-Ptyr-EO micelles (**b**); the changes of HA-coated amino-PEG-Ptyr-EO micelles with different mass ratio of HA to amino-PEG-Ptyr-EO micelles (**c**); the changes of HA-coated amino-PEG-Ptyr-EO micelles with different concentration of HA (**d**); size distribution and morphology of HA-coated amino-PEG-Ptyr-EO micelles with 0.5 mg/mL HA and 3/1 mass ratio of HA to amino-PEG-Ptyr-EO micelles (**e**); SIM release from SPMs and SPEMs in pH 7.4 PBS with or without 10 mM H_2_O_2_ (**f**). *SIM* simvastatin, *HA* hyaluronic acid, *SPEMs* SIM-loaded amino-PEG-Ptyr-EO micelles, *SHPEMs* SIM-loaded HA-coated amino-PEG-Ptyr-EO micelles, *SPMs* SIM-loaded Mal-PEG-Ptyr micelles, *SPEMs* SIM-loaded amino-PEG-Ptyr-EO micelles

Before preparing micelles, the CMC of Mal-PEG-Ptyr and Mal-PEG-Ptyr-EO was determined using pyrene as a fluorescence probe. Notably, Mal-PEG-Ptyr-EO displayed a CMC of 3.4 μg/mL, lower than that of Mal-PEG-Ptyr (8.9 μg/mL) as shown in Fig. 1a, which was possibly caused by the modification of ethyl oxalyl chloride to improve the hydrophobicity of Ptyr moieties. Micelles based on amine-PEG-Ptyr-EO were prepared by dialysis method. The size distribution and morphology were detected by DLS and TEM in Fig. 1b. It showed an average size of 131.5 ± 6.4 nm with PDI 0.106 ± 0.012. Besides, the micelles exhibited Zeta potential of 38.4 ± 9.7 mV, resulting from free amino groups. HA with molecular weight of 40–100 kDa was coated on the surface of micelles by electrostatic interaction with amino groups. As shown in Fig. 1c, the size increased and the Zeta potential decreased at the beginning with the addition of HA. However, the size decreased rapidly and the Zeta potential continued to decrease when the mass ratio of HA to amine-PEG-Ptyr-EO micelles increased to 3/1, which indicated the complete coating of HA. To explore the influence of the concentration of HA, micelles were coated with HA with concentration ranging from 0.5 to 2 mg/mL. As shown in Fig. 1d, with the increase of HA concentration, the average size of coated micelles gradually increased, while the Zeta potential was almost unchanged. Figure 1e showed the size distribution and morphology of HA-coated micelles with the mass ratio of HA (0.5 mg/mL) to micelles of 3/1. It demonstrated uniform spherical micelles with size around 142.3 nm.

### Drug loading and release

During the formation of micelles, SIM and the hydrophobic segments of PEG-Ptyr-EO were forced to aggregate by H_2_O molecules. Thus, loaded drugs enlarged the core of the micelles. The average size of SHPEMs was higher than that of HPEMs (Additional file 1: Figure S5A), indicating successful encapsulation of SIM. After SIM was loaded into micelles, the average size of SHPEMs was measured in PBS (pH 7.4) and DMEM containing 10% FBS. As shown in Additional file 1: Figure S5B, SHPEMs can remain stable in PBS and DMEM for at least half month, indicating great colloidal stability. The DLC and DLE of SPEMs were calculated as 7.3% and 84.3%, respectively. The in vitro drug release profiles of SHPEMs and SPMs in pH 7.4 PBS were evaluated by the dialysis method. As depicted in Fig. 1f, sustained release of SIM from SPEMs or SPMs can be maintained for over 72 h. Fast SIM release occurred within initial 24 h. It was noteworthy that the release profiles of SIM from SPMs and SPEMs without H_2_O_2_ addition were quite similar. Notably, SIM release from SPEMs with H_2_O_2_ addition was obviously faster, which was possibly caused by the oxidation of peroxalate esters. Specifically, the accumulative release amount of SIM from SPEMs with H_2_O_2_ addition was 54.3% for 72 h, which it was 30.6% without H_2_O_2_ addition. According to the profile of drug release, it can be concluded that SIM was gradually release from micelles and no obvious “sudden release” phenomenon was observed, indicating SIM was mainly encapsulated in the hydrophobic core of micelles. After incubation with H_2_O_2_ for 72 h, the size distribution and morphology of SHPEMs were measured as shown in Additional file 1: Figure S6. SHPEMs became anomalous aggregates with widened size distribution, which was possibly caused by the oxidation of peroxalate esters and drug release.

### Cellular uptake and cytotoxicity

**Fig. 2:**
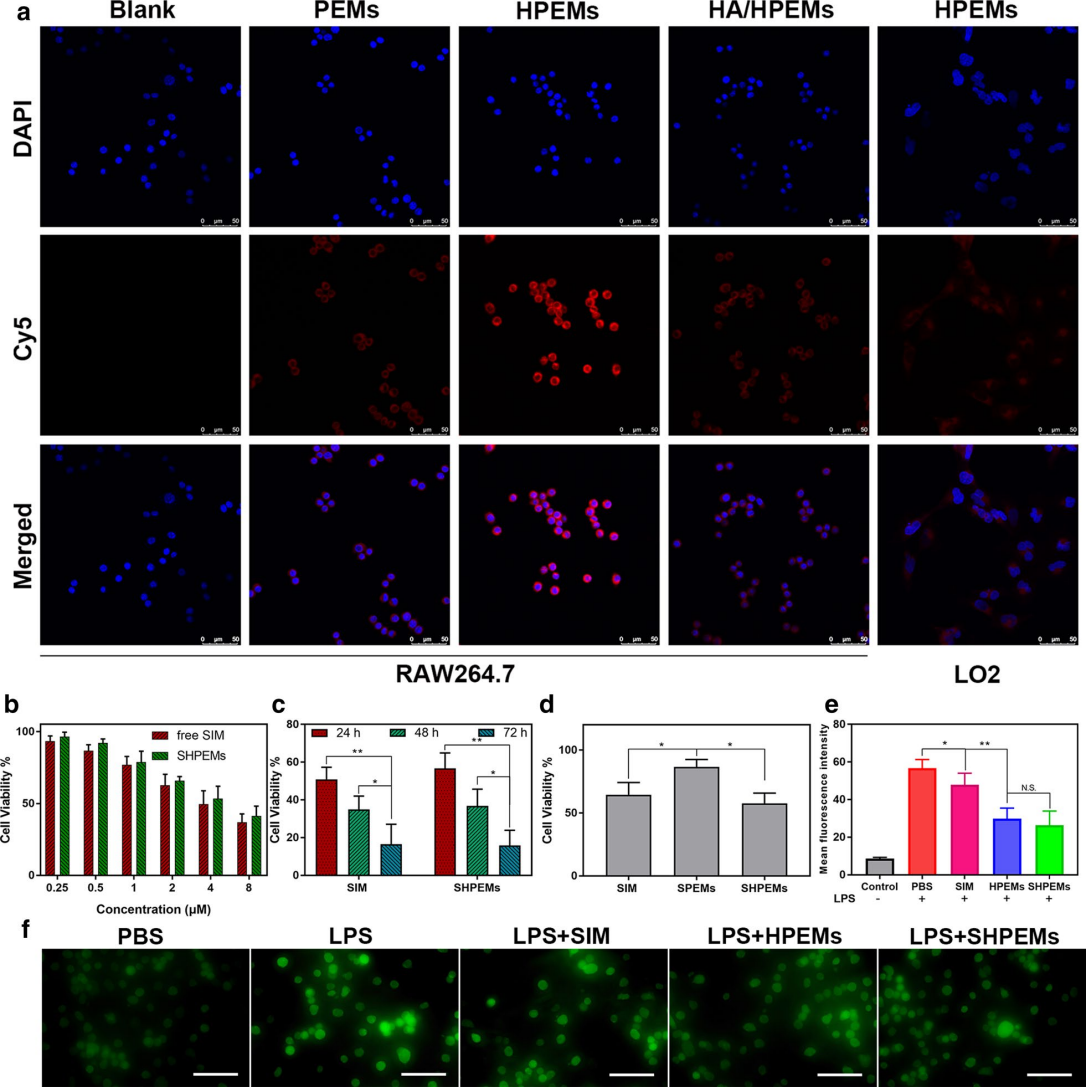
CLSM images of RAW264.7 cells treated with PBS, Cy5-labeled PEMs and HPEMs with or without HA pretreatment and LO2 cells treated with Cy5-labeled HPEMs. Scale bar: 50 μm (**a**); cell viability of free SIM and SHPEMs against RAW264.7 for 24 h (**b**); cell viability of free SIM and SHPEMs with 4 μg/mL SIM for 24 h, 48 h and 72 h (**c**); cell viability of free SIM, SPEMs and SHPEMs (8 μg/mL SIM) for 4 h (**d**); quantitative analyses for ROS production of LPS-induced RAW264.7 cells treated with PBS, SIM, HPEMs and SHPEMs by flow cytometry (**e**) and cellular imaging. Scale bar: 100 μm (**f**). *SIM* simvastatin, *HA* hyaluronic acid, *LPS* lipopolysaccharide, *SPEMs* SIM-loaded amino-PEG-Ptyr-EO micelles, *SHPEMs* SIM-loaded HA-coated amino-PEG-Ptyr-EO micelles, *HPEMs* HA-coated amino-PEG-Ptyr-EO micelles

To investigate CD44-mediated cellular uptake, LPS-induced RAW264.7 and LO2 (less CD44 expression) were incubated with different micelle formulations. Micelles were labeled with Cy5. As shown in Additional file 1: Figure S7, labeling with Cy5 hardly changed the size distribution and morphology of PEMs. As shown in Fig. 2a, both RAW264.7 and LO2 cells exhibited significant Cy5 fluorescence after treated with different micelles. Notably, cells treated with Cy5-labeled HPEMs exhibited stronger fluorescence than Cy5-labeled PEMs. However, after cells were pre-treated with free HA and then incubated with Cy5-labeled HPEMs, the red fluorescence decreased significantly. Moreover, LO2 exhibited weak Cy5 fluorescence after treatment with Cy5-labeled HPEMs. These results indicated the CD44-mediated endocytosis of LPS-induced RAW264.7 against HA-coated micelles.

To study the cytotoxicity of SIM-loaded micelles, the cell viability was tested by MTT assay. Firstly, both PEMs and HPEMs exhibited the cell viability over 90% at all tested concentrations, indicating great biocompatibility (Additional file 1: Figure S8). Both free SIM and SHPEMs showed comparative cell viability of 24 h’s incubation against RAW264.7 cells. Free SIM showed slightly higher cytotoxicity than SHPEMs at all tested SIM concentrations (Fig. 2b). Interestingly, the cell viabilities of both free SIM and SHPEMs against LO2 cells were over 90%, indicating great biosafety (Additional file 1: Figure S9). To survey time-dependent cytotoxicity, LPS-induced RAW264.7 cells were incubated free SIM and SHPEMs with 4 μg/mL SIM for 24 h, 48 h and 72 h, respectively. As shown in Fig. 2c, for SIM and SHPEMs, the cytotoxicity increased with the prolongation of culture time. To further investigate the influence of HA on the cytotoxicity, cells were incubated with free SIM, SPEMs and SHPEMs at 8 μg/mL for 4 h. After another 20 h’s incubation with normal medium, the cell viability was tested and the results were shown in Fig. 2d. Specifically, the cell viability of free SIM was 64.1%, slightly higher than that of SHPEMs (57.8%) and significantly lower than that of SPEMs (86.7%). These results suggested that HA coating on micelles facilitated cellular uptake and subsequent cytotoxicity.

To investigate the antioxidant effect, cells were treated with different formulations and intracellular ROS was detected using DCFH-DA as the probe. RAW264.7 and LPS-induced RAW264.7 treated with PBS were used as negative and positive control, respectively. As shown in Fig. 2e and Additional file 1: Figure S10, ROS increased significantly after cells were induced by LPS. After LPS-induced RAW264.7 cells were treated with free SIM, ROS decreased slightly. Notably, ROS decreased dramatically after cells were treated with HPEMs and SHPEMs. Similar results can be observed from DCF fluorescence images in Fig. 2f. These results implied that HPEMs can effectively consume intracellular ROS.

### Mice treatment

**Fig. 3:**
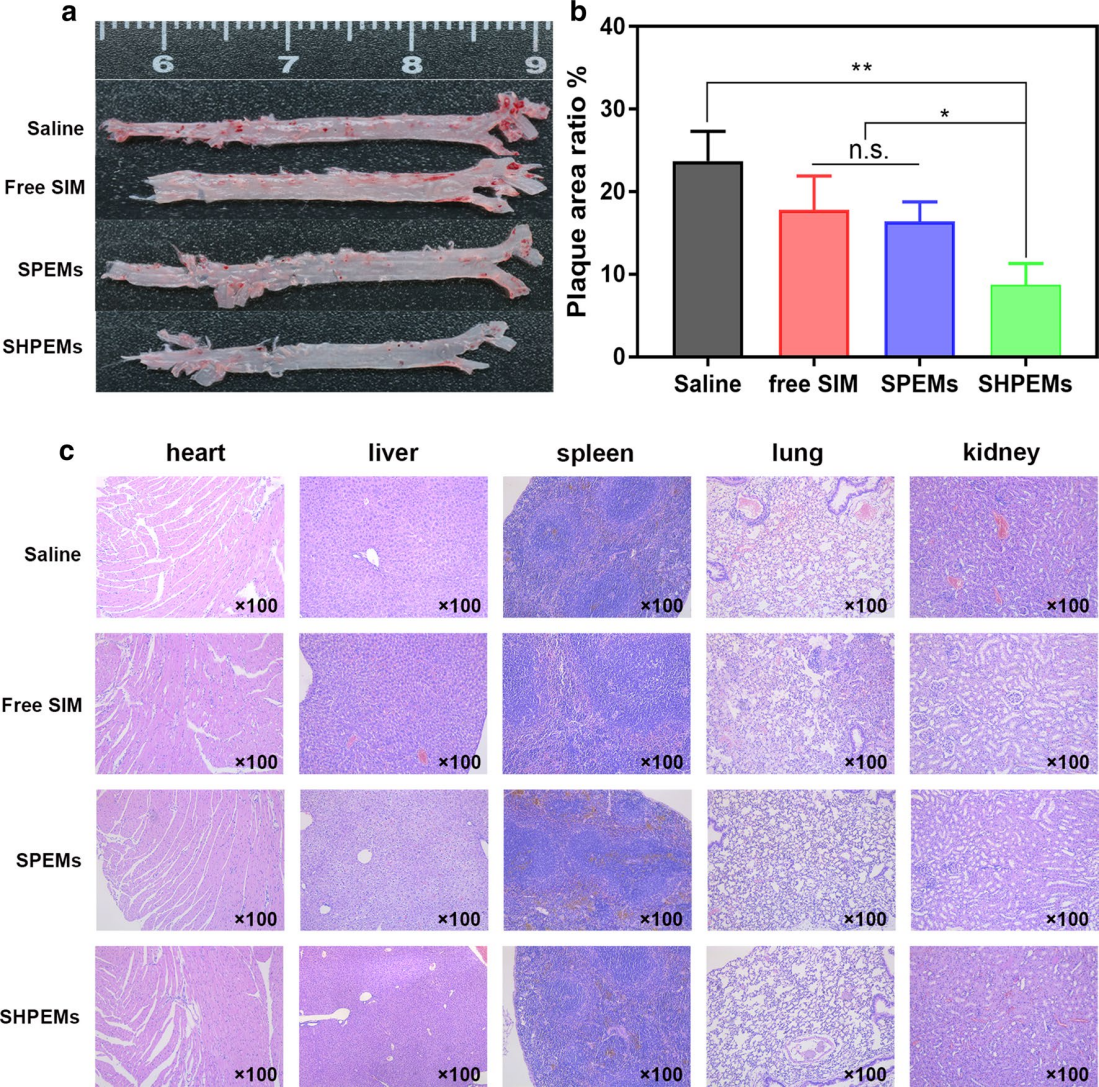
Representative photographs of ORO stained aortas from each group (**a**); the ratio of plaque area to total area (**b**); H&E staining for heart, liver, spleen, lung as well as kidney collected from mice of each group (**c**). *SIM* simvastatin, *SPEMs* SIM-loaded amino-PEG-Ptyr-EO micelles, *SHPEMs* SIM-loaded HA-coated amino-PEG-Ptyr-EO micelles

Inspired by excellent cellular uptake profiles and the ideal cytotoxicity against macrophages, in vivo research was carried out to evaluate the therapeutic efficacy of SHPEMs. AopE^−/−^ mice were fed with a high-fat diet for 8 weeks and then randomly divided into 4 groups (n = 4). Free SIM, SPEMs and SHPEMs were administrated through tail veil for another 4 weeks. Saline was administrated as the control group. After the experiment, the mice were sacrificed and the aortas and major organs were collected. The isolated aorta was stained with ORO shown in Fig. 3a and the plaque area ratio was measured as shown in Fig. 3b. The lesion area ratio of the control group with saline treatment intervention was significantly larger than that of other treatment groups. The lesion area ratio of free SIM and SPEMs was obviously smaller than the control group, indicating some therapeutic effects. Interestingly, the lesion area ratio of the SHPEMs group was significantly smaller than that of other groups, indicating enhanced anti-atherosclerotic effect. The enhanced therapeutic efficacy of SHPEMs was mainly resulted from the targeting ability derived from HA coating. Besides, the histological tissues slides of main organs in different groups were subjected to H&E staining and displayed in Fig. 3c. No obviously pathological changes can be observed in heart, liver, spleen, lung and kidney, suggesting great biosafety and biocompatibility.

## Conclusion

In conclusion, oxidative stress and recruitment of pro-inflammatory monocytes are both critical for the development of atherosclerotic plaques in the aorta. SIM possesses excellent anti-inflammatory activity, whereas the poor water solubility and low bioavailability limit its application in vivo. Therefore, an amphipathic block copolymer PEG-Ptyr-EO with oxidation sensitivity was synthesized, assembled into SIM-loaded micelles and coated with HA for targeted atherosclerosis treatment. The SIM-loaded HA-coated micelles showed excellent atherosclerotic plaque-targeted property. The in vitro tests proved that SHPEMs effectively suppressed the growth of RAW264.7 cells and significantly reduced the oxidative stress in macrophages. On the other hand, by reacting with H_2_O_2_, the micellar carrier itself further alleviated oxidative stress in LPS-induced RAW264.7. Consequently, the CD44-targeted and H_2_O_2_-responsive polymeric micelles loaded with SIM possess excellent therapeutic effects and low side effects in atherosclerosis treatment.

## Supplementary Information

**Additional file 1.** Additional figures.

## Data Availability

The datasets used and/or analyzed during the current study are available from the corresponding author on reasonable request.

## Abbreviations

SIM: Simvastatin
HA: Hyaluronic acid
PEG-Ptyr-EO: Poly(ethylene glycol)-poly(tyrosine-ethyl oxalyl)
ROS: Reactive oxygen species
LDL: Low-density lipoprotein
CVD: Cardiovascular disease
Mal-PEG-NH_2_: Maleimide poly(ethylene glycol) amine
Tyr-NCA: L-Tyrosine *N*-carboxyanhydride
LPS: Lipopolysaccharide
DCFH-DA: 2′, 7′-Dichlorofluorescin diacetate
DMF: *N*, *N*-Dimethylformamide
DCM: Dichloromethane
DMSO: Dimethylsulfoxide
THF: Tetrahydrofuran
TEA: Triethylamine
GPC: Gel permeation chromatography
CMC: Critical micelle concentrations
PBS: Phosphate buffer solution
PEMs: Amino-PEG-Ptyr-EO micelles
HPEMs: HA-coated PEMs
TEM: Transmission electron microscopy
SHPEMs: SIM-loaded HPEMs
DLE: Drug loading efficiency
DLC: Drug loading content
SPEMs: SIM-loaded PEMs
SPMs: SIM-loaded Mal-PEG-Ptyr micelles
ORO: Oil red O
