# Supplementary material for: Hyaluronic acid-coated polymeric micelles with hydrogen peroxide scavenging to encapsulate statins for alleviating atherosclerosis
Source: J Nanobiotechnology. 2020 Dec 7;18:179. doi: 10.1186/s12951-020-00744-w (PMC7720571; doi:10.1186/s12951-020-00744-w)
Supplement: Supplementary file 1 — Additional file 1. Additional figures. [file 12951_2020_744_MOESM1_ESM.docx]

**Hyaluronic acid-coated polymeric micelles with hydrogen peroxide scavenging to encapsulate statins for alleviating atherosclerosis**

Dan Mu^a *^, Jianhui Li^b^, Yu Qi^c^, Xuan Sun^c^, Yihai Liu^b^, Song Shen^b^, Yuyu Li^d^, Biao Xu^c e *^, Bing Zhang^a f *^

^a^Department of Radiology, Affiliated Nanjing Drum Tower Hospital of Nanjing University Medical School, Nanjing, 210008, China.

^b^Department of Cardiology, Nanjing Drum Tower Hospital, Clinical College of Nanjing Medical University, Nanjing, 210008, China.

^c^Department of Cardiology, Affiliated Nanjing Drum Tower Hospital of Nanjing University Medical School, Nanjing, 210008, China.

^d^Department of Cardiology, Nanjing Drum Tower Hospital, Medical School of Nanjing University, Nanjing, 210008, China.

^e^State Key Laboratory of Pharmaceutical Biotechnology, Nanjing University, Nanjing, 210023, Jiangsu, China.

^f^Institute of Brain Science, Nanjing University, Nanjing, Jiangsu, 210008, China.

*Correspondence to:

Dan Mu, Department of Radiology, Affiliated Nanjing Drum Tower Hospital of Nanjing University Medical School, Nanjing, 321 Zhongshan road, Nanjing, Jiangsu, China, 210008 (e-mail: [mudan118@126.com](mailto:mudan118@126.com)).

Biao Xu, Department of Cardiology, Affiliated Nanjing Drum Tower Hospital of Nanjing University Medical School, Nanjing, 210008, China. State Key Laboratory of Pharmaceutical Biotechnology, Nanjing University, 321 Zhongshan road, Nanjing, Jiangsu, China, 210008 (e-mail: [xubiao62@nju.edu.cn](mailto:xubiao62@nju.edu.cn)).

Bing Zhang, Department of Radiology, Affiliated Nanjing Drum Tower Hospital of Nanjing University Medical School, Nanjing, 210008, China. Institute of brain Science, Nanjing University, 321 Zhongshan road, Nanjing, Jiangsu, China, 210008 (e-mail: [zhangbing_nanjing@nju.edu.cn](mailto:zhangbing_nanjing@nju.edu.cn)).


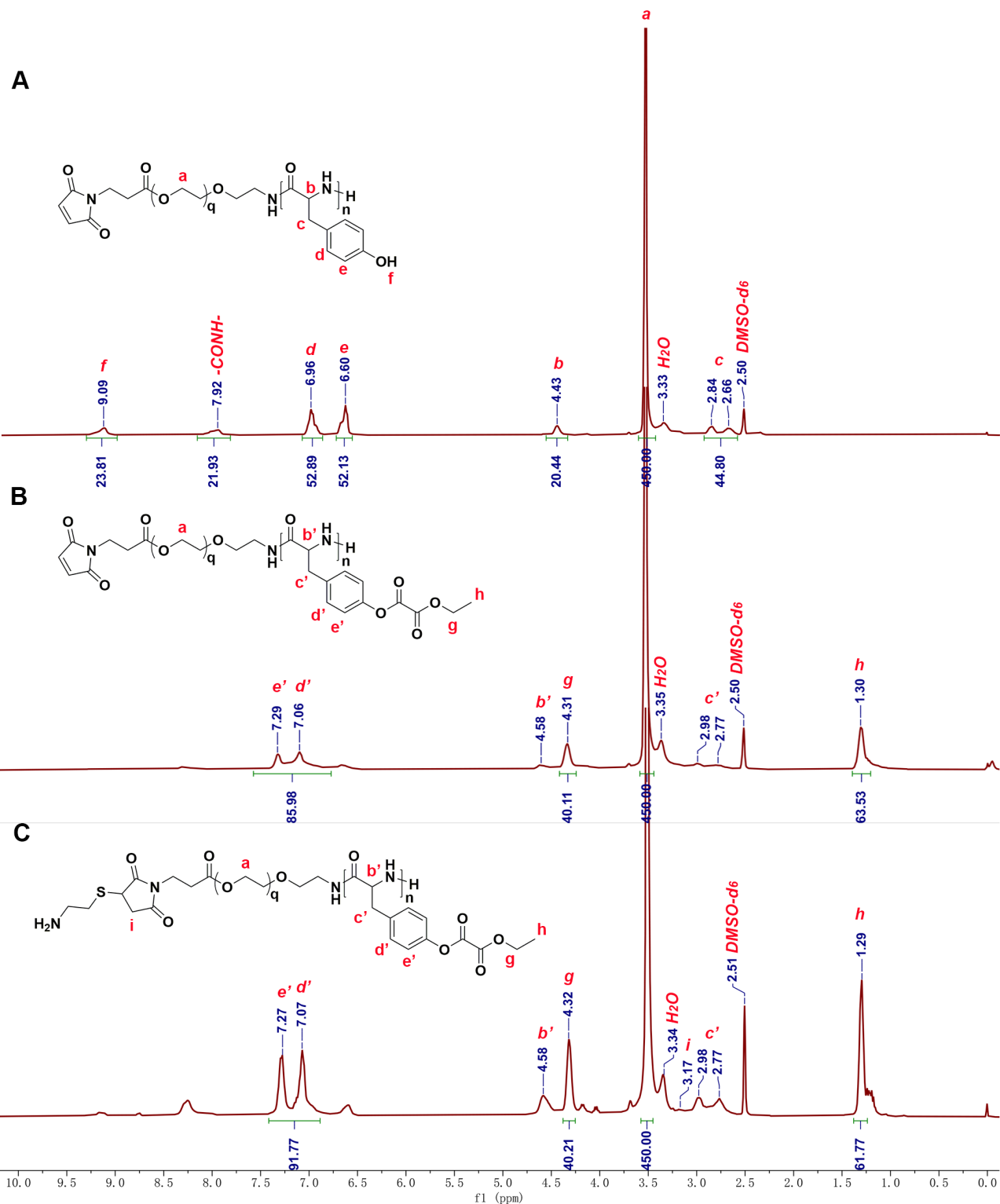


**Figure S1.** ^1^H-NMR (500 MHz) of the Mal-PEG-Ptyr in DMSO-*d*_6_ (A), Mal-PEG-Ptyr-EO in DMSO-*d*_6_ (B) and amino-PEG-Ptyr-EO in DMSO-*d*_6_ (C).


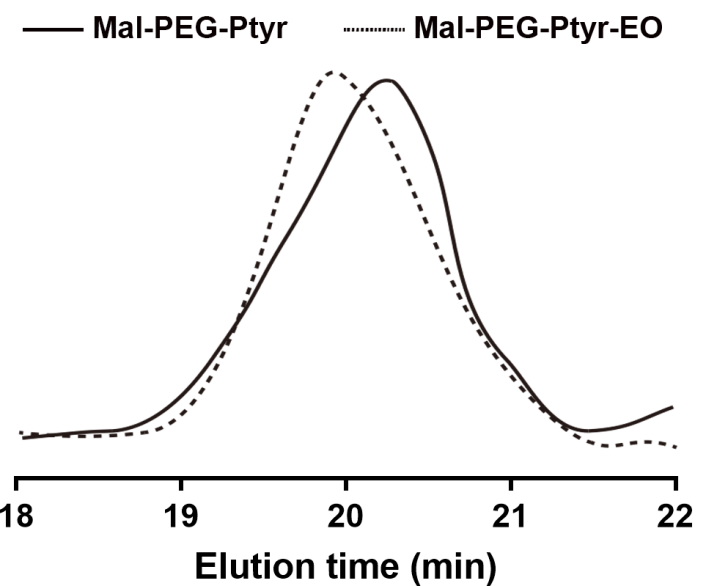


**Figure S2.** The GPC traces of Mal-PEG-Ptyr and Mal-PEG-Ptyr-EO.


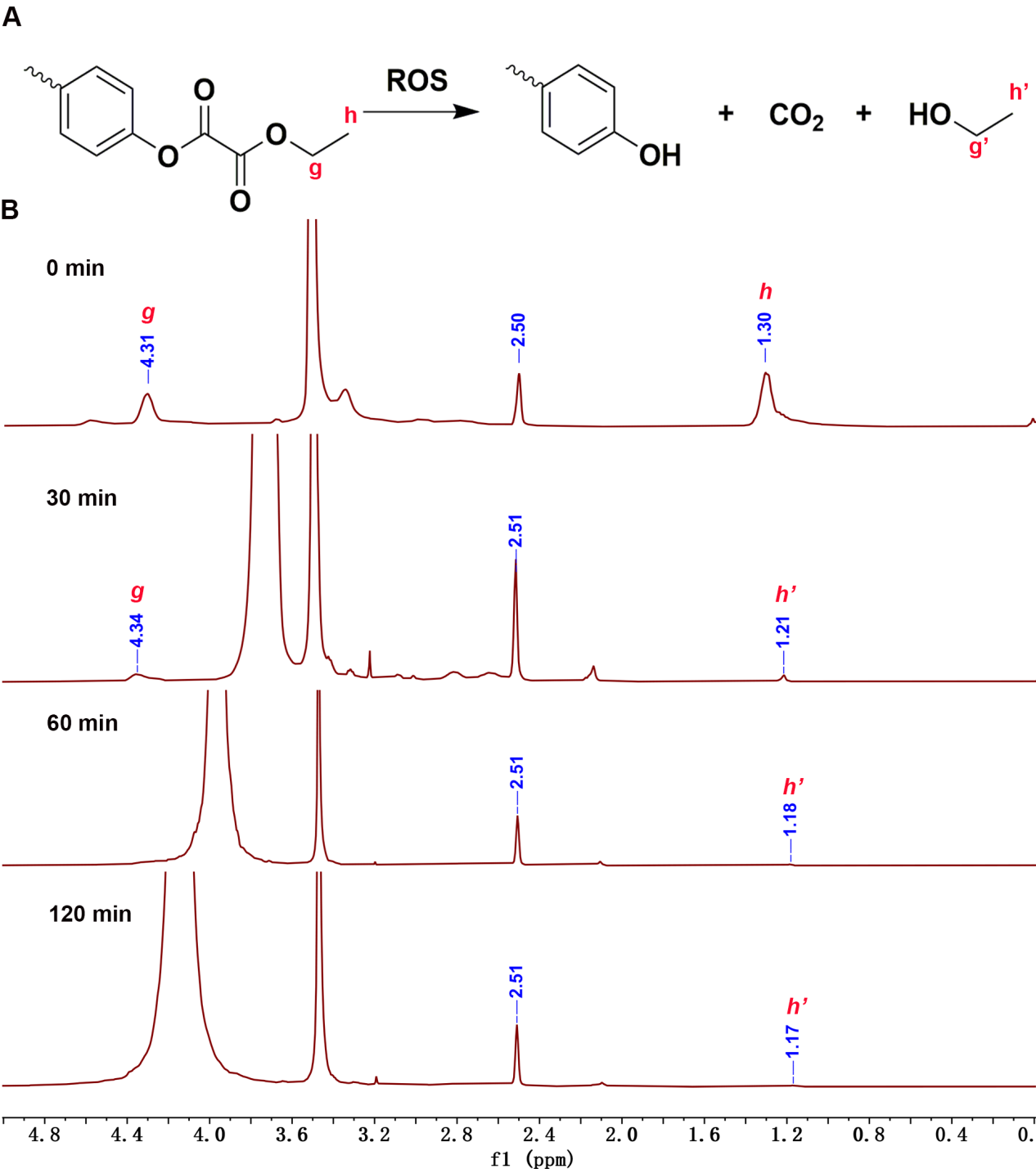


**Figure S3.** Illustration of the oxidation of peroxalate ester (A); structural changes of Mal-PEG-Ptyr-EO treated with 50 mM H_2_O_2_ for 30 min, 60 min and 120 min (B).


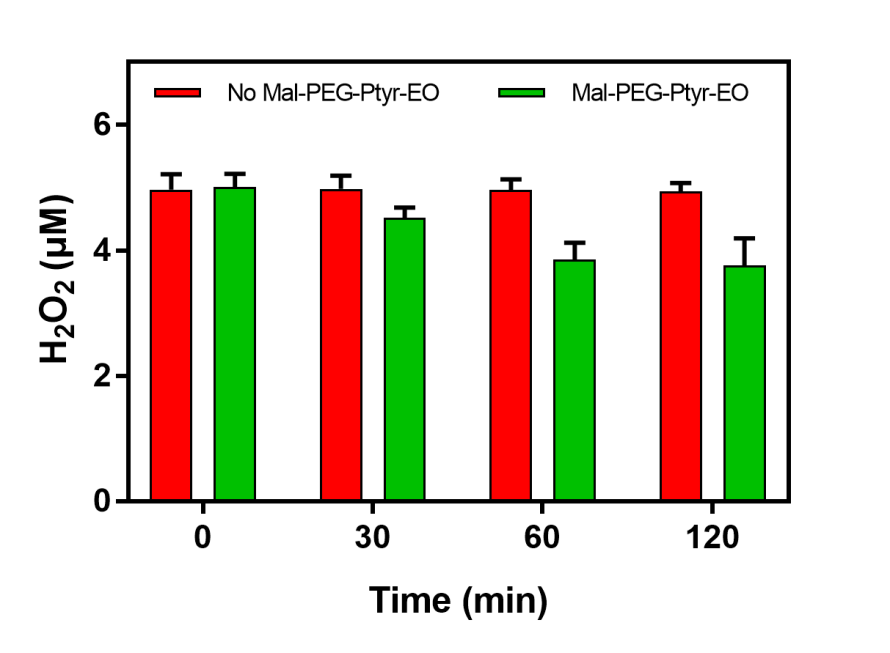


**Figure S4.** The amount of H_2_O_2_ after incubation with Mal-PEG-Ptyr-EO.


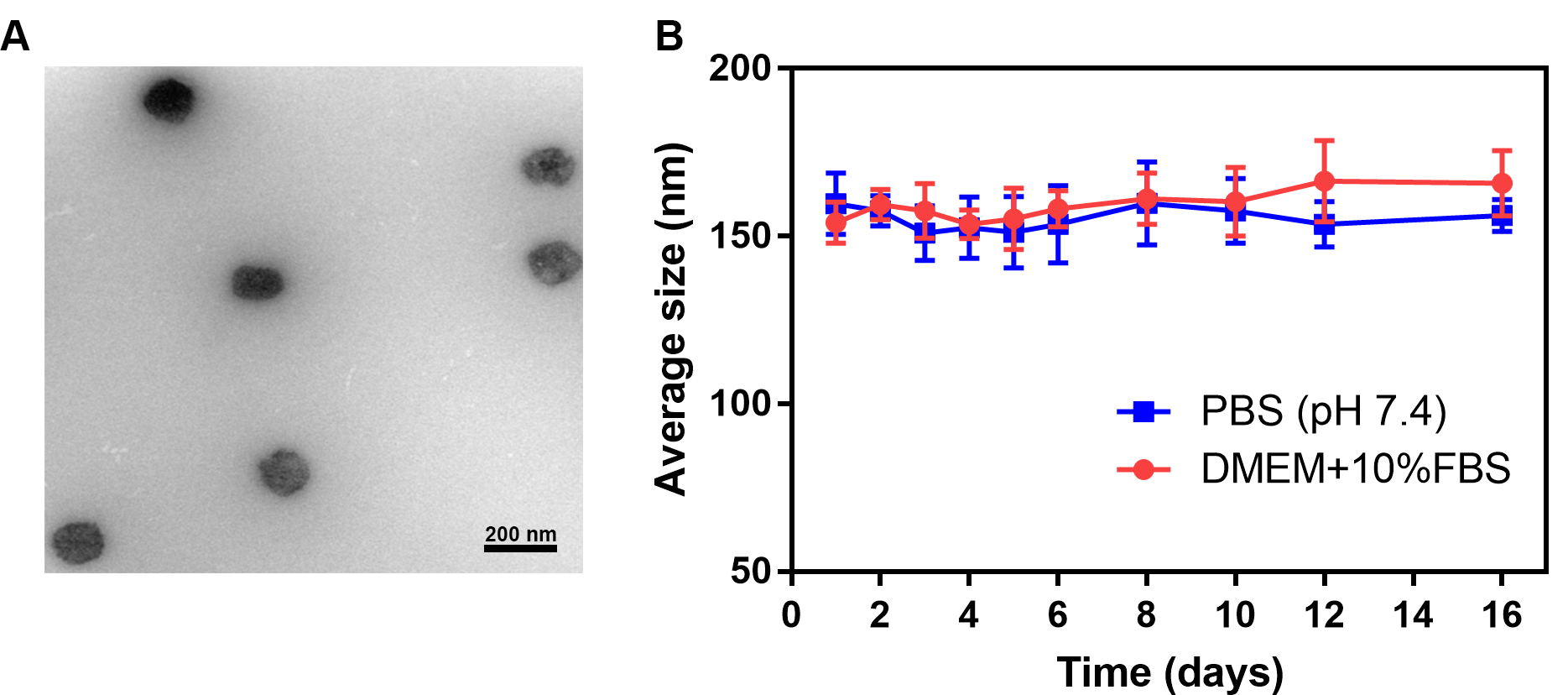


**Figure S5.** The morphology of SHPEMs (A); the colloidal stability of SHPEMs in PBS (pH 7.4) and DMEM with 10 % FBS (B).


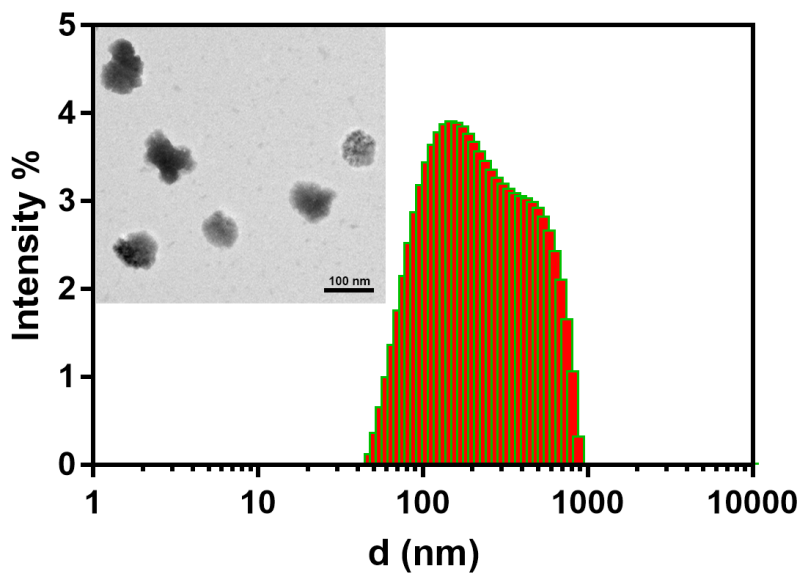


**Figure S6.** Size distribution and morphology of SHPEMs after treated with H_2_O_2_ for 72 h.


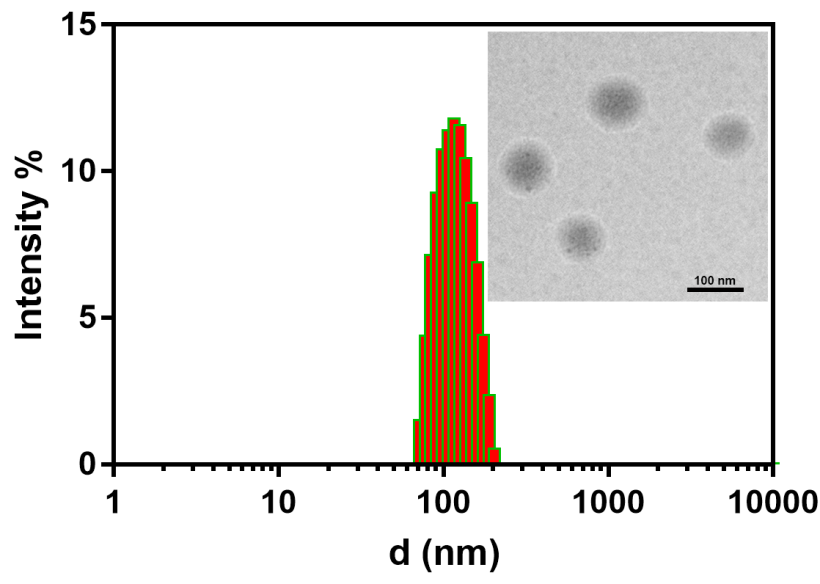


**Figure S7.** The size distribution and morphology of Cy5-labeled PEMs.


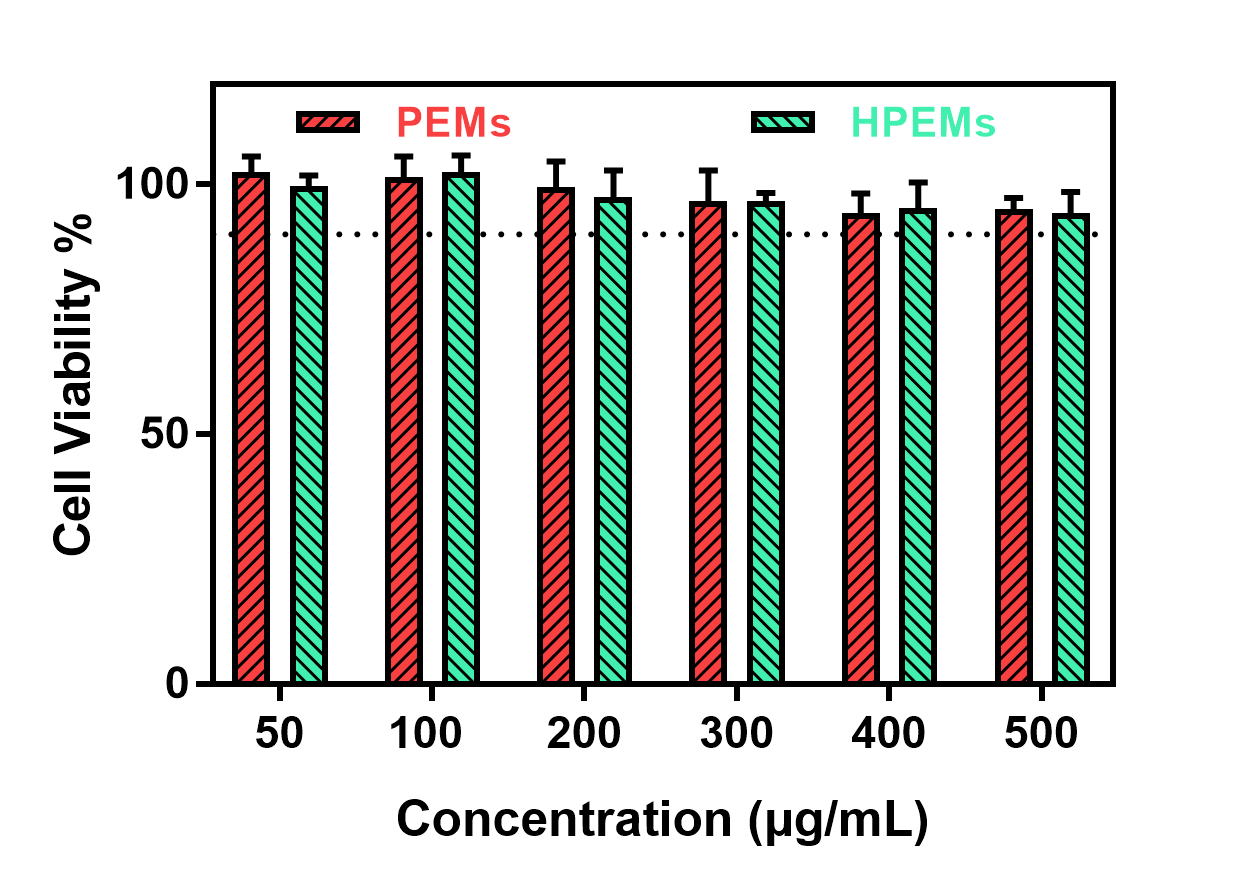


**Figure S8.** Cytotoxicity of LPS-induced RAW264.7 macrophage cells incubated with PEMs and HPEMs for 24 h.


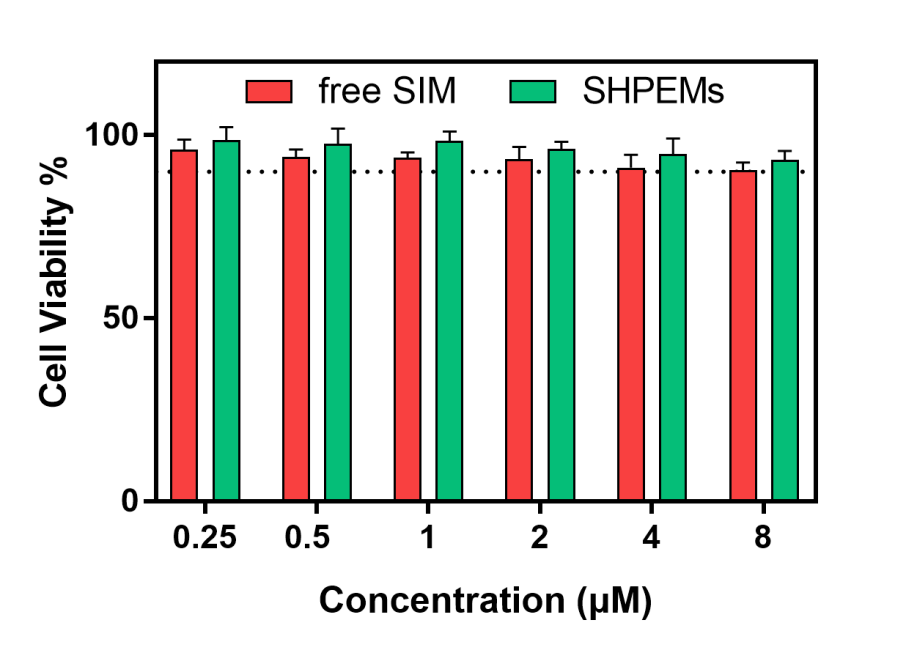


**Figure S9.** Cell viability of free SIM and SHPEMs against LO2 cells for 24 h.


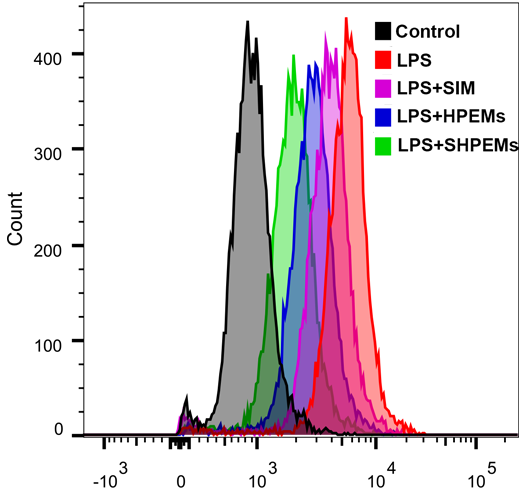


**Figure S10.** Flow cytometry analysis of ROS generation after treatment with different formulations.
